# The role of wildlife (wild birds) in the global transmission of antimicrobial resistance genes

**DOI:** 10.24272/j.issn.2095-8137.2017.003

**Published:** 2017-03-18

**Authors:** Jing Wang, Zhen-Bao Ma, Zhen-Ling Zeng, Xue-Wen Yang, Ying Huang, Jian-Hua Liu

**Affiliations:** College of Veterinary Medicine, South China Agricultural University, Guangzhou 510642, China; College of Veterinary Medicine, South China Agricultural University, Guangzhou 510642, China; College of Veterinary Medicine, South China Agricultural University, Guangzhou 510642, China; College of Veterinary Medicine, South China Agricultural University, Guangzhou 510642, China; College of Veterinary Medicine, South China Agricultural University, Guangzhou 510642, China; College of Veterinary Medicine, South China Agricultural University, Guangzhou 510642, China

**Keywords:** AmpC, ESBLs, IMP, *mcr-1*, NDM, Wild birds

## Abstract

Antimicrobial resistance is an urgent global health challenge in human and veterinary medicine. Wild animals are not directly exposed to clinically relevant antibiotics; however, antibacterial resistance in wild animals has been increasingly reported worldwide in parallel to the situation in human and veterinary medicine. This underlies the complexity of bacterial resistance in wild animals and the possible interspecies transmission between humans, domestic animals, the environment, and wildlife. This review summarizes the current data on expanded-spectrum β-lactamase (ESBL), AmpC β-lactamase, carbapenemase, and colistin resistance genes in *Enterobacteriaceae* isolates of wildlife origin. The aim of this review is to better understand the important role of wild animals as reservoirs and vectors in the global dissemination of crucial clinical antibacterial resistance. In this regard, continued surveillance is urgently needed worldwide.

## INTRODUCTION

Over several decades, antimicrobial resistance has become a global clinical and public health threat against the effective treatment of common infections caused by resistant pathogens, resulting in treatment failure and increased mortality (WHO, 2014). The development of bacterial resistance is a natural evolution of microorganisms, but the widespread use and misuse of antibacterial agents in humans and animals has accelerated this process (WHO, 2014). Furthermore, the increasing frequency of global travel and trade has also contributed to the rapid worldwide spread of antimicrobial resistance (Laxminarayan et al., 2013). Some resistant clones, such as *Escherichia coli* ST131, *Klebsiella pneumoniae* ST258 and ST11, and methicillin-resistant *Staphylococcus aureus* (MRSA) USA 300, which are involved in the spread of resistance to crucially significant antibiotics in human medicine, have been widely disseminated (Lee et al., 2016; Mathers et al., 2015; Nimmo, 2012). Antimicrobial resistance is a complex and multifaceted problem involving humans, animals, and the environment. However, the role of wildlife in the emergence of antibacterial resistance might be underestimated. The first report of antibacterial resistance in wildlife revealed chloramphenicol resistance in *E. coli* isolates obtained from Japanese wild birds (Sato et al., 1978). Since then, the occurrence of resistant bacteria in wild animals has been increasingly reported within diverse animal species across different geographical areas. In addition, several important antimicrobial resistant pathogens, such as MRSA (Loncaric et al., 2013a; Porrero et al., 2014), vancomycin-resistant enterococci (Drobni et al., 2009; Sellin et al., 2000), *Salmonella* spp. (Lee et al., 2011a), *Vibrio cholerae* (Aberkane et al., 2015), and *Campylobacter* spp. (Weis et al., 2016), have been described in wild animals, highlighting the importance and complexity of wildlife, not normally exposed to antibiotics directly, in the transmission of resistant bacteria.

This review gives a brief overview of the emergence and prevalence of expanded-spectrum β-lactamase (ESBL), AmpC β-lactamase, carbapenemase, and colistin resistance genes in *Enterobacteriaceae* strains from wild animals, all of which have significant public health impact. Furthermore, this review aims to better understand the role of wildlife in the transmission of clinically significant antimicrobial resistance in *Enterobacteriaceae*.

## ESBL-PRODUCING *ENTEROBACTERIACEAE* FROM WILDLIFE

The global dissemination of ESBL-producing *Enterobacteriaceae* in human clinics is an urgent problem that poses a serious challenge to the treatment of infectious diseases, particularly the worldwide emergence of CTX-M-15-producing ST131 *E. coli* (Alghoribi et al., 2015; Blanco et al., 2013; Hansen et al., 2014; Hussain et al., 2014; Mathers et al., 2015; Platell et al., 2011; Sauget et al., 2016). ESBL-producing *Enterobacteriaceae* have also been increasingly reported in livestock, companion animals, and food (Aliyu et al., 2016; Braun et al., 2016; Ewers et al., 2010; Hordijk et al., 2013; Michael et al., 2016). The CTX-M-type β-lactamases are the most common ESBLs among *Enterobacteriaceae* isolates of human and veterinary origin worldwide (Hordijk et al., 2013; Liu et al., 2016a; Pietsch et al., 2017; Wang et al., 2016; Wellington et al., 2013).

Since the first report on ESBL-producing *E. coli* isolates from wild animals in Portugal in 2006 (Costa et al., 2006), ESBL-producing *Enterobacteriaceae* of wildlife origin have so far been reported in Europe, Africa, Asia, South America, North America, and Australia (Table 1). Although ESBLs have been found in various *Enterobacteriaceae*, most ESBL-producing bacterial pathogens in wild animals are *E. coli*, followed by *K. pneumoniae* (Table 1). To date, at least 80 wildlife species have been found to be carriers of ESBL-producing *Enterobacteriaceae*, most being wild birds (Table 1). Similar to that among isolates from human and veterinary medicine, the CTX-M family is the most prevalent type of ESBL-producing *Enterobacteriaceae* found in wild animals (Table 1). Both *bla*_CTX-M-1_ and *bla*_CTX-M-15_ are commonly reported in wild animals and are the most prevalent ESBL genes, followed by *bla*_CTX-M-14_, *bla*_CTX-M-32_, *bla*_CTX-M-9_, *bla*_CTX-M-3_, *bla*_CTX-M-2_, and *bla*_CTX-M-22_. Other ESBL genes, such as *bla*_CTX-M-27_, *bla*_CTX-M-55_, *bla*_CTX-M-8_, *bla*_CTX-M-24_, *bla*_CTX-M-25_, *bla*_CTX-M-28_, *bla*_CTX-M-29_, and *bla*_CTX-M-124_, have also been detected, though infrequently (Table 1). Significant geographical differences have been observed in the occurrence of CTX-M enzymes. As summarized in Table 1, CTX-M-15 is the only reported CTX-M-type β-lactamase in Africa to date, and is the most common CTX-M-type enzyme reported in Bangladesh. In Canada and the US, CTX-M-14 is dominant, followed by CTX-M-15. Diversity in CTX-M β-lactamases has been reported in European countries, with the predominance of CTX-M-1 and CTX-M-15. Interestingly, CTX-M-15 is also reported to be the most common CTX-M enzyme in Franklin's gulls (*Leucophaeus pipixcan*) in northern Chile (Báez et al., 2015), although CTX-M-1 was previously reported to be dominant in the same gull species in central Chile (Hernandez et al., 2013). Báez et al. (2015) hypothesized that, based on their migratory habits, Franklin's gulls from the north acquired resistant CTX-M-15-producing ST131 and ST10 *E. coli* clones, which are highly prevalent in humans in the US and Canada but scarce in Chile. However, this hypothesis, though possible, needs further investigation.

**Table 1:** Presence of extended-spectrum β-lactamase (ESBL)-producing Enterobacteriaceae in wild animals

Animal species	Year of sampling	Species	No. of ESBL-producing isolates	Detected ESBL types (no.)	Plasmid replicon typing^a^	Insertion sequence	MLST	Country	References
Bird of prey, Deer, Fox, Owl (all unspecified)	2003-2004	*E. coli*	9	*bla*_CTX-M-14_ (3) *bla*_CTX-M-14_+*bla*_TEM-52_ (1) *bla*_CTX-M-1_ (1) *bla*_SHV-12_ (1) *bla*_TEM-52_ (3)		IS*Ecp1*- *bla*_CTX-M-14_(4), IS*Ecp1*- *bla*_CTX-M-1_		Portugal	Costa et al., 2006
Common kestrel, Sparrow hawk,	2003-2004	*E. coli*	2	*bla*_CTX-M-14_ (1) *bla*_CTX-M-14_ + *bla*_TEM-52_ (1)				Portugal	Costa et al., 2008
Seagulls (not specified)	2007	*E. coli*	11	*bla*_TEM-52_ (8) *bla*_CTX-M-1_(1) *bla*_CTX-M-14_(1) *bla*_CTX-M-32_(1)		IS*Ecp1*- *bla*_CTX-M1_, IS*Ecp1*-IS*5*- *bla*_CTX-M132_, IS*Ecp1*-*bla*_CTX-M14_		Portugal	Poeta et al., 2008
Yellow-legged gulls (*Larus michahellis*)	Not specified	*E. coli*	16	*bla*_CTX-M-1_ (7) *bla*_CTX-M-1_+*bla*_TEM_ (1)^*^ bla_CTX-M-15_+*bla*_TEM_ (1)^*^ bla_SHV_+*bla*_TEM_ (2)^*^ bla_TEM_ (5)^*^			ST90, ST156, ST351, ST533, ST681 (2), ST746, ST1134, ST1135, ST1140 (2), ST1142, ST1143 (2), ST1144, ST1199	France	Bonnedahl et al., 2009
Black-headed gull (*Larus ridibundus*)	2006	*E. coli*	7	*bla*_CTX-M-1_ (1) *bla*_CTX-M-15_(2) *bla*_SHV-2_(1) *bla*_SHV-12_(2)				Czech Republic	Dolejská et al., 2009
Wild boars	2005-2007	*E. coli*	8	*bla*_CTX-M-1_(8)				Portugal	Poeta et al., 2009
Common buzzards (*Buteo buteo*)	2007-2008	*E. coli*	10	*bla*_CTX-M-32_(7) *bla*_CTX-M-1_(3)		IS*Ecp1*-*bla*_CTX-M-1_(3)		Portugal	Radhouani et al., 2010
Black-headed gull (*Larus ridibundus*)	2008	*E. coli*	3	*bla*_CTX-M-14_ (2) *bla*_CTX-M-15_+*bla*_TEM_(1)^*^			ST1340, ST1646, ST1647	Sweden	Bonnedahl et al., 2010
Glaucous winged gull (*Larus glaucescens*)	2007	*E. coli*	4	*bla*_CTX-M-14_ (1) *bla*_CTX-M-14_+*bla*_TEM_(1) *bla*_CTX-M-15_+*bla*_TEM_ (2)^*^			ST131, ST609 (2), ST746	Russia	Hernandez et al., 2010
Mallard, Herring gull, Waterbird	2008-2009	*E. coli*	9	*bla*_CTX-M-1_ (6) *bla*_CTX-M-9_ (1) *bla*_CTX-M-15_ (1) *bla*_SHV-12_ (1)	~35 kb IncN (*bla*_CTX-M-1_) ~90 kb IncI1 (*bla*_CTX-M-1_) ~80 kb NT (*bla*_CTX-M-1_) ~100 kb IncI1 (*bla*_CTX-M-1_) ~95 kb IncI1 (*bla*_SHV-12_)			Poland	Literak et al., 2010a
Wild boar (*Sus scrofa*)	2006-2007	*E. coli*	5	*bla*_CTX-M-1_ (4) *bla*_TEM-52b_ (1)	~90 kb IncI1 (*bla*_CTX-M-1_)(4) ~45 kb NT (*bla*_TEM-52b_)			Czech Republic, Slovakia	Literak et al., 2010b
Eurasian blackbirds (*Turdus merula*), Rock pigeon (*Columba livia*), Greater white-fronted goose (*Anser albifrons*)	2006	*E. coli*	4	*bla*_CTX-M-15_ (4)	> 100 kb IncFII-FIA-FIB		ST648 (4)	Germany	Guenther et al., 2010a
Brown rat (*Rattus norvegicus*)	Not specified	*E. coli*	1	*bla*_CTX-M-9_ (1)	> 100 kb FIA-FIB		ST131	Germany	Guenther et al., 2010b
Common buzzard (*Buteo buteo*), Common barn owl (*Tyto alba*), Eurasian tawny owl (*Strix aluco*), Booted eagle (*Hieraaetus pennatus*), Montagu's harrier (*Circus pygargus*), Black kite (*Milvus migrans*), Eurasian black vulture (*Aegypius monachus*), Bonelli's eagle (*Hieraaetus fasciatus*), Eurasian eagle owl (*Bubo bubo*), Common raven (*Corvus corax*)	2008	*E. coli*	32	*bla*_CTX-M-1_(27) *bla*_CTX-M-1_+ *bla*_TEM-20_ (1) *bla*_SHV-5_(3) *bla*_TEM-20_ (1)				Portugal	Pinto et al., 2010
Seagulls (*Larus fuscus*, *Larus cachinnans*)	2007-2008	*E. coli*	45	*bla*_CTX-M-1_ (8) *bla*_TEM-9_ (4) *bla*_CTX-M-15_ (17) *bla*_CTX-M-32_ (15)			ST10, ST43, ST58, ST69, ST86, ST131 (4), ST155, ST156, ST165, ST205, ST224 (3), ST297, ST359, ST405, ST453, ST559, ST1152, ST1163, ST1284 (4)	Portugal	Simões et al., 2010
Canada goose (*Branta canadensis*), Wild domestic goose (*Anser anser domesticus*)	2010	*E. coli*	2	*bla*_SHV-12_(1) *bla*_TEM-52_ (1)			ST1079 ST1844	Belgium	Garmyn et al., 2011
Iberian lynx (*Lynx pardinus*)	2008-2010	*E. coli*	10	*bla*_CTX-M-14_ (6) *bla*_SHV-12_(4)		IS*Ecp1*-*bla*_CTX-M14_(6)		Spain	Gonçalves et al., 2012
Wild rodents	2008-2010	*E. coli*	19	*bla*_CTX-M-1_(9) *bla*_CTX-M-9_(8)				Hong Kong, China	Ho et al., 2011
Blackcap (*Sylvia atricapilla*)	2006-2010	*E. coli*	1	*bla*_CTX-M-14_ + *bla*_SHV-12_ (1)				Azores Archipelago/ Portugal	Silva et al., 2011
Gilthead Seabream (*Sparus aurata*)	2007	*E. coli*	5	*bla*_TEM-52_(2) *bla*_SHV-12_(3)				Atlantic Ocean, West coast of Portugal	Sousa et al., 2011
Black-headed gull (*Larus ridibundus*), Common gull (*Larus canus*), Herring gull (*Larus argentatus*), Lesser black backed gull (*Larus fuscus*)	2010	*E. coli*	18	*bla*_CTX-M-1_(16) *bla*_CTX-M-14_ (1) *bla*_SHV-12_(1)				Sweden	Wallensten et al., 2011
Black kites (*Milvus migrans*), Red kites (*Milvus milvus*), Buzzard (*Buteo buteo*)	2010	*E. coli*	9	*bla*_CTX-M-1_ (9)			ST12, ST744 (4), ST847, ST1640, ST2199, ST2198	Germany	Guenther et al., 2012
Black kites (*Milvus migrans*), Black vultures (*Aegypius monachus*), Demoiselle cranes (*Anthropoides virgo*)	2010	*E. coli*	5	*bla*_CTX-M-9_ (4) *bla*_CTX-M-55_ (1)			ST117, ST167, ST648 (2), ST2346	Mongolia	
Common teal (*Anas crecca*), Tufted duck (*Aythya fuligula*)	2009	*E. coli*	3	*bla*_CTX-M-15_ (3)			ST1312, ST1408 (2)	Bangladesh	Hasan et al., 2012
Seagull (*Larus delawarensis*), Pelican	2010	*E. coli*	8	*bla*_CTX-M-15_ (5) *bla*_CTX-M-32_ (2) *bla*_CTX-M-124_ (1)	FIA-FIB (*bla*_CTX-M-15_) (4), FIB (*bla*_CTX-M-15_) (1)		ST10, ST405, ST410, ST559, ST617, ST648, ST853, ST1845	Florida, USA	Poirel et al., 2012
		*E. cloacae*	2	*bla*_SHV-7_(2)	IncL/M (2)				
Roe, Deer	2011	*E. coli*	1	*bla*_CTX-M-1_(1)				Switzerland	Stephan & Hächler, 2012
Great cormorants (*Phalacrocorax carbo*)	2006-2007, 2009	*E. coli*	10	*bla*_CTX-M-15_ (7) *bla*_CTX-M-27_ (2)	170kb FIA-FIB (*bla*_CTX-M-15_)(1) 100 kb IncI1 (*bla*_CTX-M-15_) (2) 120 kb FIA-FIB (*bla*_CTX-M-27_)(1)	IS*Ecp1*-*bla*_CTX-M_(9)	ST131 (2)	Czech Republic, Slovak Republic	Tausova et al., 2012
Franklin's gulls (*Leucophaeus pipixcan*)	2009	*E. coli*	129^b^	*bla*_CTX-M-1_(39) *bla*_CTX-M-15_(8) *bla*_CTX-M-14_(1) *bla*_CTX-M-3_(2)			ST10, ST34, ST38, ST48, ST54, ST58, ST69, ST70, ST127, ST131, ST155, ST167, ST216, ST349, ST367, ST405, ST617, ST641, ST847, ST1106, ST1711, ST1712, ST1714, ST1715, ST1722, ST1723, ST1724, ST1716, ST1713, ST1725	Chile	Hernandez et al., 2013
Rooks (*Corvus frugilegus*)	2013	*E. coli*	20	*bla*_CTX-M-1_ (15) *bla*_CTX-M-3_ (3) *bla*_CTX-M-15_ (3) *bla*_TEM-15_(1)	IncI1-IncIγ (*bla*_CTX-M-1_) (3), IncN (*bla*_CTX-M-1_) (3), IncF-FIB-IncI1-Iγ (*bla*_TEM-15_), IncF-FIB-IncI1-Iγ-N (*bla*_CTX-M-1_) (2), IncF-FIB-IncN (*bla*_CTX-M-1_) (2), IncHI1 (*bla*_CTX-M-1_) (3), IncF-FIA-FIB (*bla*_CTX-M-15_) (3), IncF (*bla*_CTX-M-3_)	IS*Ecp1*-*bla*_CTX-M_(18)	ST23, ST34 (2), ST58(2), ST69 (2), ST90, ST131, ST162, ST491, ST744 (3), ST1683(5)	Austria	Loncaric et al., 2013b
Red fox (*Vulpes vulpes*)	2008-2009	*E. coli*	2	*bla*_SHV-12_(2)			ST1086	Portugal	Radhouani et al., 2013
European herring gull, Mallard, Black-headed gull, Egyptian goose, Feral pigeon, Grey heron, Mute swan, Northern gannet, Northern lapwing, Gadwall, Common redshank, Lesser black-backed gull, Tufted duck, Common gull, Common goldeneye, Common guillemot, Black swan, Barnacle goose, Ruff	2010-2011	*E. coli*	65	*bla*_CTX-M-1_ (14) *bla*_CTX-M-15_ (13) *bla*_CTX-M-3_ (8) *bla*_CTX-M-14_ (8) *bla*_CTX-M-32_ (3) *bla*_SHV-12_ (3) *bla*_TEM-52c_ (1) *bla*_SHV-12_ *+bla*_TEM-52c_ (1)	IncI1(*bla*_CTX-M-1_) (13), IncF (*bla*_CTX-M-1_), IncB/O (*bla*_CTX-M-14_), IncF (*bla*_CTX-M-14_) (6), IncF (*bla*_CTX-M-15_) (3), IncHI2 (*bla*_CTX-M-15_), IncHI2-IncF (*bla*_CTX-M-15_), IncI1 (*bla*_CTX-M-15_) (4), IncI2 (*bla*_CTX-M-15_), IncF (*bla*_CTX-M-3_)(6), IncI1 (*bla*_CTX-M-3_), IncF (*bla*_CTX-M-32_), IncF (*bla*_SHV-12_), IncI1 (*bla*_SHV-12_), IncN (*bla*_SHV-12_), IncI1(*bla*_SHV-12_*+bla*_TEM-52c_), IncI1 (*bla*_TEM-52c_)			Netherlands	Veldman et al., 2013
Gulls (unspecified)	2010	*E. coli*	33	*bla*_CTX-M-14_ (23) *bla*_CTX-M-27_ (3) *bla*_CTX-M-15_ (1) *bla*_TEM-19_ (5)			ST10 (1), ST38 (10), ST131 (12), ST405 (3), ST2253, ST2967 (6)	Barrow, Alaska, USA	Bonnedahl et al., 2014
		*K. pneumoniae*	35	*bla*_CTX-M-15_ (1) *bla*_CTX-M-14_ (23) *bla*_TEM-19_ (1) *bla*_TEM-52_ (1) *bla*_SHV-2_ (1) *bla*_SHV-12_ (7) *bla*_SHV-102_ (8) *bla*_SHV-102_+ *bla*_TEM-19_ (2) *bla*_CTX-M-15_ (1)+ *bla*_SHV-1_ (4) *bla*_CTX-M-15_ (1)+ *bla*_SHV-12_ (4)					
*Boops boops*, *Sardina pilchardus*, *Sarpa salpa, Trachurus trachurus, Pagellus acarne*	2012-2013	*E. coli*	22	*bla*_CTX-M-15_(15) CTX-Mgroup 9 (1) *bla*_TEM-24_(6)			ST8, ST21, ST31 (2), ST37, ST66, ST74, ST131 (2), ST132, ST398 (3), ST471 (7), ST477 (2)	Mediterranean Sea	Brahmi et al., 2015
Brown-headed gull (*Chroicocephalus brunnicephalus*)	2010	*E. coli*	29	*bla*_CTX-M-15_ (28) *bla*_CTX-M-14_ (1)			ST10, ST48, ST131, ST345, ST349, ST648, ST853, ST1727, ST2687, ST2688, ST2689,	Bengal	Hasan et al., 2014
		*K. pneumoniae*	1	*bla*_CTX-M-15_ (1)					
		*E. albertii*	1	*bla*_CTX-M-55+_ *bla*_CTX-M-79_(1)					
Wintering rooks (*Corvus frugilegus*)	2011	*E. coli*	82	*bla*_CTX-M-1_ (39) *bla*_CTX-M-8_ (1) *bla*_CTX-M-14_ (2) *bla*_CTX-M-15_ (25) *bla*_CTX-M-24_ (4) *bla*_CTX-M-25_ (1) *bla*_CTX-M-28_ (1) *bla*_CTX-M-55_ (2) *bla*_TEM-52_(4) *bla*_SHV-12_(2)			ST10 (7), ST48 (3), ST58 (8), ST88, ST101, ST106, ST115 (4), ST131 (11), ST154, ST155, ST167(3), ST206, ST351, ST361, ST394, ST398 (2), ST405, ST448, ST542, ST609, ST617 (5), ST641, ST669, ST746 (3), ST1011, ST1141, ST1249, ST1638 (2), ST1642, ST1725, ST1832 (2), ST2226, ST3014 (3), ST3015, ST3017, ST3018, ST3019, ST3020, ST3056, ST3223	Czech Republic, Germany, Poland, Serbia, Spain	Jamborova et al., 2015
Franklin's gulls (*Leucophaeus pipixcan*)	2011	*E. coli*	67	*bla*_CTX-M-15_ (38) *bla*_CTX-M-2_ (12) *bla*_CTX-M-22_ (11) *bla*_CTX-M-3_ (1) *bla*_TEM-40_ (5) *bla*_TEM-198_ (1)			ST10 (6), ST38 (6), ST44 (10), ST131 (12), ST205, ST350 (3), ST359, ST405 (2), ST540 (2), ST617 (6), ST642, ST648, ST665 (4), ST744, ST2277, ST3476, ST4184, ST4185, ST4186, ST4187 (3), ST4188, ST4189	Chile	Báez et al., 2015
Franklin's gulls (*Leucophaeus pipixcan*)	2010	*E. coli*	55^c^	*bla*_CTX-M-1_(2) *bla*_CTX-M-3_(1) *bla*_CTX-M-14_(23) *bla*_CTX-M-15_(16) *bla*_CTX-M-55_(1) *bla*_CTX-M-14_+ *bla*_TEM-52_(1)			ST10, ST12, ST38 (5), ST48, ST69 (5), ST131, ST167 (3), ST617, ST1304, ST1431	Canada	Bonnedahl et al., 2015
		*K. pneumoniae*	7	*bla*_CTX-M-14_(1) *bla*_CTX-M-15_(3) *bla*_SHV-2_(1), *bla*_SHV-2A_(1) *bla*_SHV-11_(1) *bla*_SHV-12_(1) *bla*_SHV-14_(1)					
House crow (*Corvus splendens*)	2010	*E. coli*	154^d^	*bla*_CTX-M-1_ *bla*_CTX-M-14_ *bla*_CTX-M-15_ *bla*_CTX-M-55_(142) *bla*_CTX-M-79_			ST38, ST46, ST58 (2), ST131 (6), ST354 (3), ST405, ST1664, ST1706, ST2141, ST2521, ST3486, ST3487, ST3488, ST3135, ST3482 (2), ST3780, ST3781, ST3782	Bangladesh	Hasan et al., 2015
		*K. pneumoniae*	21	*bla*_CTX-M-15_(17)					
		*E. cloacae*	4	*bla*_CTX-M-15_(3)					
		*Citrobacter freundii*	3	*bla*_CTX-M-15_(3)					
		*Proteus mirabilis*	1	*bla*_CTX-M-14_					
		*Raoultella terrigena*	14	*bla*_CTX-M-15_(14)					
		*Escherichia spp.*	1	*bla*_CTX-M-15_(1)					
Common kestrel (*Falco tinnunculus*), Rock dove (*Columba livia*), Baboon monkey (*Papio hamadryas*), Yemen linnet (*Carduelis yemenensis*), African gray parrot (*Psittacus erithacus*)	Not specified	*E. coli E. cloacae K. pneumonia K. oxytoca Citrobacter freundii*	5	*bla*_CTX-M_(5)				Saudi Arabia	Hassan & Shobrak, 2015
Brown rats, Black rats, Indo-Chinese forest rats	2008-2013	*E. coli*	77	*bla*_CTX-M-1_(36) *bla*_CTX-M-9_(31) *bla*_CTX-M-1_+ *bla*_CTX-M-9_ (2)			ST10 (6), ST12, ST34, ST38 (4), ST48, ST58 (2), ST69 (3), ST75, ST88, ST117 (5), ST131 (2), ST155 (6), ST156, ST162, ST224 (4), ST226, ST366, ST405 (2), ST410, ST414, ST602, ST617, ST641, ST648 (2), ST994, ST1081, ST1380, ST2003, ST2165, ST2178, ST2253, ST2913, ST3489, ST3894, ST4375, ST4531, ST4532, ST4533, ST4534, ST4535, ST4537, ST5436	Hong Kong, China	Ho et al., 2015
Herring gulls (*Larus argentatus*), Lesser-black backed gulls (*Larus fuscus*), Yellow-legged gulls (*Larus michahellis*)	2009	*E. coli K. pneumoniae K. oxytoca Citrobacter spp. Enterobacter spp.*	948^e^	M1 group (*bla*_CTX-M-1_ *bla*_CTX-M-3_*bla*_CTX-M-15_*bla*_CTX-M-32_ *bla*_CTX-M-55_) (318), M2 group (*bla*_CTX-M-2_)(28), M8 group (*bla*_CTX-M-8_) (1), M9 group (*bla*_CTX-M-9_ *bla*_CTX-M-14_ *bla*_CTX-M-24_ *bla*_CTX-M-27_ *bla*_CTX-M-65_) (254), *bla*_SHV_(372), *bla*_TEM_(222)				England, Ireland, Latvia, Poland, Portugal, Spain, Sweden, Netherlands	Stedt et al., 2015
Open bill stork (*Anastomus oscitans*)	2010	*E. coli*	2	*bla*_CTX-M-15_ (2)			ST2689, ST4016	Bangladesh	Rashid et al., 2015
White stork (*Ciconia ciconia*), Black kite (*Milvus migrans*), Red kite (*Milvus milvus*), Golden eagle (*Aquila chrysaetos*), Griffon vulture (*Gyps fulvus*), Common cuckoo (*Cuculus canorus*), Barn owl (*Tyto alba*), Spotless starling (*Sturnus unicolor*)	2013-2014	*E. coli*	14	*bla*_CTX-M-1_ (3) *bla*_CTX-M-14_ (2) *bla*_SHV-12_(9)			ST10, ST38, ST57, ST131, ST155, ST156, ST453 (3), ST744, ST877, ST1158, ST1431, ST3778	Spain	Alcalá et al., 2016
Glaucous-winged gulls (*Larus glaucescen*s), Herring gulls (*Larus argentatus*)	2014	*E. coli*	3	*bla*_CTX-M-15_ (3)				Alaska, USA	Atterby, et al., 2016
Wild boars, Barbary macaques	2014-2015	*E. coli*	30	*bla*_CTX-M-15_(30)			ST69 (2), ST131 (3), ST226 (2), ST405 (3), ST617 (13), ST648 (4), ST1421 (2), ST1431	Algeria	Bachiri et al., 2017
		*K. pneumoniae*	17	*bla*_CTX-M-15_(17)			ST584 (17)		
Wild boar, Gull, Rook (not specified)	Not specified	*E. coli*	8	*bla*_SHV-12_(2) *bla*_TEM-52b_(2) *bla*_TEM-176_(4)	40 kb IncX1, 45 kb IncX1 45 kb IncX2, 55 kb IncX2 145 kb IncX2-I1 (2) 50 kb IncX3 80 kb IncX3-N			Australia, Czech Republic, Germany	Dobiasova & Dolejska, 2016
Cattle egret, Black vulture, Pigeon	2012	*E. coli*	10	*bla*_CTX-M-15_ (6) *bla*_CTX-M-32_ (2) *bla*_CTX-M-2_ (1) *bla*_CTX-M-22_ (1)				Nicaragua	Hasan et al., 2016
Kelp gull (*Larus dominicanus*)	2012	*E. coli*	5	*bla*_CTX-M-14_ (4) *bla*_CTX-M-2_ (1)			ST101, ST744 (4)	Argentina	Liakopoulos et al., 2016
Mouflons (*Ovis orientalis musimon*)	2013	*E. coli*	1	*bla*_CTX-M-15_ (1)	FIA-FIB		ST744	Austria	Loncaric et al., 2016
		*K. pneumoniae*	1	*bla*_SHV-11_(1)			ST11		
		*Klebsiella spp.*	1	*bla*_CTX-M-3_ (1)					
Crow	2014	*E. coli*	3	*bla*_CTX-M-15_ (1) *bla*_SHV-2_(2)				Canada	Parker et al., 2016
Mute swan (*Cygnus olor*), Sea eagle, Crow, Goshawk, Buzzard, Herring gull, Sparrowhawk, Pigeon, Bean goose, Magpie, Grey heron, Marsh harrier	2011-2014	*E. coli*	24	*bla*_CTX-M-1_ (13) *bla*_CTX-M-55_ (8)			ST88, ST115, ST131, ST167, ST224, ST373, ST398, ST405 (2), ST410 (3), ST617 (3), ST648, ST1167, ST1204, ST1304, ST1670, ST1730, ST1968, ST4306, ST4307	Germany	Schaufler et al., 2016
		*E. cloacae*	1	*bla*_CTX-M-1_ (1)					

* TEM and SHV genotype not specified. ^a^ Plasmid replicons performed on original isolates are not listed in Table 1; NT, plasmid replicons not determined by PCR-based replicon typing. ^b^ 50 of 129 ESBL-producing isolates were randomly selected for further genotype and MLST analysis. ^c^ 20 of 55 ESBL-producing *E. coli* isolates were selected for MLST analysis. ^d^ Numbers of *bla*_CTX-M-1_, *bla*_CTX-M-14_, *bla*_CTX-M-15_, and *bla*_CTX-M-79_ were not specified; 27 of 154 ESBL-producing isolates were selected for MLST analysis. ^e^ Numbers of *bla*_TEM_ and *bla*_SHV_, including *bla*_TEM-1_, *bla*_TEM-2_, and *bla*_SHV-1_, which were not true ESBLs.

In addition to CTX-M enzymes, SHV and TEM enzymes have also been reported in wildlife, especially SHV-12 and TEM-52, which accords with that found in ESBL-producing isolates from humans, livestock, and companion animals (Table 1) (Blanco et al., 2013; Carattoli et al., 2005; Hordijk et al., 2013; Michael et al., 2016; Smet et al., 2010). For example, SHV-12 has been frequently detected in wildlife in Spain (Alcalá *et al.*, 2016; Gonçalves *et al.*, 2012), and is highly prevalent in ESBL-producing *E. coli* obtained from 8-to 16-month-old healthy children in northern Spain (Fernández-Reyes et al., 2014) and in raw poultry meat from southern Spain (Egea et al., 2012), as well as from hospitals (Blanco et al., 2013). Other SHV-type enzymes, such as SHV-102, SHV-1, SHV-2, and SHV-5, and TEM-type enzymes, such as TEM-19, TEM-40, TEM-176, and TEM-20, have also been sporadically reported in wild animals (Table 1).

More than 170 different sequence types (STs) have been identified in ESBL-producing *E. coli* isolates of wildlife origin (Table 1). Among them, ST131 is the most commonly detected clone. The dominant ST131 clone identified in wild animals, which has been frequently described in humans, companion animals, food products, and the environment, is involved in the international dissemination of *bla*_CTX-M-15_ and *bla*_CTX-M-14_ (Alghoribi et al., 2015; Bogaerts et al., 2015; Ewers et al., 2010; Hu et al., 2013; Hussain et al., 2014; Kawamura et al., 2014; Kim et al., 2017; Mathers et al., 2015). Additionally, other STs described in wild animals, such as ST10, ST69, ST405, ST410, and ST648, have also been reported in various sources and are responsible for the intercontinental distribution of CTX-M (Fischer et al., 2014, 2017; Hansen et al., 2014; Hu et al., 2013; Liu et al., 2016a; Müller et al., 2016; Su et al., 2016; Wang et al., 2016). However, some STs found in wildlife, such as ST1340, ST1646, ST2687, ST3018, and ST3056, have been identified as new types and have not yet been reported in human or veterinary isolates (Bonnedahl *et al.*, 2010; Hasan *et al.*, 2014; Jamborova *et al.*, 2015).

As for ESBL-producing *K. pneumoniae*, limited studies are currently available on the clonal group of *K. pneumoniae* from wildlife (Table 1). Loncaric et al. (2016) found an SHV-11-encoding *K. pneumoniae* strain from mouflon (*Ovis orientalis musimon*) in Austria belonging to the epidemic clone ST11, which is associated with carbapenemase (Hu et al., 2016; Kim et al., 2013; Lee et al., 2016; Voulgari et al., 2016) and ESBL in humans worldwide (Hu et al., 2016; Lee et al., 2011b; Lu et al., 2016; Sennati et al., 2012), and previously described in companion animals and Eurasian beaver (*Castor fiber*) (Donati et al., 2014; Pilo et al., 2015). In Algeria, all 17 *bla*_CTX-M-15_-bearing *K. pneumoniae* isolates found in wild boars and Barbary macaques belong to ST584, which has also been detected in silver gulls as carriers of carbapenemase IMP-4 in Australia (Dolejska et al., 2016) as well as in human in Brazil (http://bigsdb.pasteur.fr/klebsiella/klebsiella.html).

Successful clones found in humans and domestic and wild animals indicate possible interspecies transmission of ESBL-producing isolates. However, horizontal transfer mediated by mobile elements, such as insertion sequences and plasmids, is also one of the main methods for ESBL dissemination worldwide (Carattoli, 2013; Partridge, 2015). Only limited (mostly European) studies are available on ESBL-encoding plasmids in wild animals (Table 1). For example, *bla*_CTX-M-15_ is reportedly associated with IncF plasmids (mostly multiple replicons containing IncFIA and IncFIB) and IncI1 (Guenther et al., 2010a; Loncaric et al., 2016; Poirel et al., 2012; Tausova et al., 2012; Veldman et al., 2013), which agrees with previous research involving CTX-M-15-producing *Enterobacteriaceae* obtained from the environment, healthy cattle, and humans (Zurfluh et al., 2015). IncHI2 plasmids have also been reported as carriers of *bla*_CTX-M-15_ in humans (Harrois et al., 2014; Nilsen et al., 2013), companion animals (Haenni et al., 2016), pigs (Tamang et al., 2015), and wild birds (Veldman et al., 2013). Though rare, IncI2 plasmid has also been described with *bla*_CTX-M-15_ in the lesser black-backed gull from the Netherlands (Veldman et al., 2013) and identified in a chicken *E. coli* strain in China (Liu et al., 2015). In *Enterobacteriaceae* of human and veterinary origin, *bla*_CTX-M-1_ has been frequently found associated with IncN and IncI1 plasmids (Carattoli, 2009; Jakobsen et al., 2015; Madec et al., 2015). Interestingly, *bla*_CTX-M-1_ has been mainly located on IncI1 plasmids in wildlife in Europe as well (Literak et al., 2010a, b; Loncaric et al., 2013b; Veldman et al., 2013). However, IncN, IncF, and IncHI1 plasmids have also been reported as carriers of *bla*_CTX-M-1_ (Literak et al., 2010a; Loncaric et al., 2013b; Veldman et al., 2013). Furthermore, IncI1 plasmids are also carriers of other ESBL genes in wild animals, such as *bla*_CTX-M-3_, *bla*_SHV-12_, and *bla*_TEM-52_ (Poirel et al., 2012; Veldman et al., 2013). Similarly, IncF plasmids are also reported to be associated with *bla*_CTX-M-3_, *bla*_CTX-M-9_, *bla*_CTX-M-14_, *bla*_CTX-M-27_, *bla*_CTX-M-32_, and *bla*_SHV-12_ in wildlife (Guenther et al., 2010b; Poirel et al., 2012; Tausova et al., 2012; Veldman et al., 2013). The narrow-host-range plasmid IncX has been found to carry several ESBL genes, namely *bla*_TEM-135_, *bla*_TEM-52b_, *bla*_TEM-176_, and *bla*_SHV-12_, in *E. coli* isolated from diverse sources in Australia, Czech Republic, Spain, and Poland (Dobiasova & Dolejska, 2016). Notably, plasmid replicon typing was performed on ESBL-producing isolates with multiple plasmids in several studies, thus the replicons of ESBL-carrying plasmids could not be confirmed (Gonçalves et al., 2012; Hernandez et al., 2013).

Insertion sequences also play an important role in facilitating the spread of ESBL genes (Partridge, 2015). Though few studies are available on horizontal transfer mediated by insertion sequences, associations of insertion sequence IS*Ecp1* and *bla*_CTX-M_, including *bla*_CTX-M-1_, *bla*_CTX-M-14_, *bla*_CTX-M-15_, *bla*_CTX-M-27_, and *bla*_CTX-M-32_, have been observed in *E. coli* from wild animals (Costa et al., 2006; Gonçalves et al., 2012; Poeta et al., 2008; Radhouani et al., 2010; Tausova et al., 2012).

In summary, the ESBL gene types identified in wild animals are the same as those in human and veterinary medicine. Thus, interspecies transmission mediated by successful pandemic ESBL-producing clones and plasmids in humans, domestic animals, and wildlife might occur.

## PLASMID-MEDIATED AmpC β-LACTAMASE-PRODUCING *ENTEROBACTERIACEAE* FROM WILDLIFE

Plasmid-mediated AmpC β-lactamases among *Enterobacteriaceae* in human and veterinary medicine are of considerable global concern because they confer resistance to clinically important cephalosporin antibiotics and β-lactamase inhibitors (Jacoby, 2009; Smet et al., 2010). CMY-2 is the most prevalent AmpC β-lactamase and has been globally disseminated among *Enterobacteriaceae* in humans, companion animals, food-producing animals, and retail meat (Bogaerts et al., 2015; Carmo et al., 2014; Hansen et al., 2016; Jacoby, 2009; Ma et al., 2012; Smet et al., 2010; Vogt et al., 2014; Wu et al., 2015). As shown in Table 2, AmpC β-lactamases have been reported in *E. coli*, *K. pneumoniae*, and *Enterobacter cloacae* isolates of wildlife origin in Europe, North America, and Asia, and particularly in central Europe, similar to ESBL-producing *Enterobacteriaceae* (Table 2). To date, 20 different wild animal species, mostly birds, have been identified as *bla*_CMY-2_ carriers (Table 2). Like that in human and veterinary medicine, *bla*_CMY-2_ is the most commonly detected AmpC-type β-lactamase among wild animals, though its identification has been limited to *E. coli* isolates.

**Table 2:** Presence of plasmid-mediated AmpC β-lactamase-producing *Enterobacteriaceae* in wild animals

Animal species	Year of sampling	Species	No. of AmpC-producing isolates	Detected AmpC types (no.)	Plasmid replicon typing (no. of isolates)	MLST (no. of isolates)	Country	References
Raccoons (*Procyon lotor*)	2007	*E. coli*	3	*bla*_CMY-2_ (3)			Canada	Jardine et al., 2012
Seagull (*Larus delawarensis*), Pelican	2010	*E. coli*	16	*bla*_CMY-2_ (16)	IncI1(11), IncFIB (1), IncF (2)	ST38 (2), ST68 (2), ST162, ST167 (2), ST224, ST540, ST617, ST963 (6),	Florida, USA	Poirel et al., 2012
		*K. pneumoniae*	1	*bla*_FOX-5_ (1)	IncA/C			
Black kite (*Milvus migrans*)	*Not specified*	*Salmonella*Corvallis	1	*bla*_CMY-16_	IncA/C		Germany	Fischer et al., 2013
Rooks (*Corvus frugilegus*)	2013	*E. coli*	2	*bla*_CMY-2_ (2)		ST224	Austria	Loncaric et al., 2013b
European herring gull, Common redshank, Great black-backed gull, Black-headed gull, Domesticated duck, Herring gull, Lesser black-backed gull, Mute swan, Mallard	2010-2011	*E. coli*	14	*bla*_CMY-2_ (14)	IncB/O, IncI1 (10), IncK (2)		Netherlands	Veldman et al., 2013
Wintering rooks (*Corvus frugilegus*)	2011	*E. coli*	56	*bla*_CMY-2_ (47)		ST10 (3), ST23, ST57, ST58 (4), ST69 (2), ST93 (2), ST95, ST117 (3), ST131, ST351 (4), ST354, ST429 (3), ST453 (3), ST615 (2), ST665, ST770, ST963 (3), ST1056, ST1167 (2), ST1431, ST3274, ST3568, ST3778, ST4274, ST4275, ST4276	Czech Republic, Poland	Jamborova et al., 2015
Glaucous gull (*Larus hyperboreus*)	2010	*E. cloacae*	1	*bla*_ACT-23_ (1)			Norway	Literak et al., 2014
Arabian red fox (*Vulpes vulpes*)	Not specified	*E. coli*	2	*bla*_CMY-2_ (1) *bla*_DHA-1_(1)			Saudi Arabia	Hassan & Shobrak, 2015
Eurasian beaver (*Castor fiber*)	2013	*K. pneumoniae*	3	*bla*_DHA-1_(3)		ST11 (3)	Switzerland	Pilo et al., 2015
Yellow-legged gull (*Larus michahellis*)	2013-2014	*E. coli*	1	*bla*_CMY-2_ (1)		ST10	Spain	Alcalá et al., 2016
Glaucous-winged gulls (*Larus glaucescens*), Herring gulls (*Larus argentatus*)	2014	*E. coli*	5	*bla*_CMY-2_ (5)			Alaska, USA	Atterby et al., 2016
Gull (unspecified)	Not specified	*E. coli*	2	*bla*_CMY-2_ (2)	50 kb IncX1 (2)		Czech Republic	Dobiasova & Dolejska, 2016
Mouflons (*Ovis orientalis musimon*)	2013	*K. pneumoniae*	1	*bla*_DHA-1_(1)		ST11	Austria	Loncaric et al., 2016

The *bla*_CMY-2_ gene is mainly located on IncA/C and IncI1 plasmids from *Enterobacteriaceae* isolates of human and veterinary origin (Bogaerts et al., 2015; Bortolaia et al., 2014; Carattoli, 2009; Guo et al., 2014; Sidjabat et al., 2014). Among wild animals, *bla*_CMY-2_ has been reported to be mainly associated with IncI1 plasmids in *E. coli* from Dutch wild birds and wild seagulls in the US (Poirel et al., 2012; Veldman et al., 2013). Other plasmid types, such as IncB/O, IncK, and IncF, have also been identified as carriers of *bla*_CMY-2_ in wildlife (Poirel et al., 2012; Veldman et al., 2013), as previously reported in humans, companion animals, broiler chickens, and retail meat (Bortolaia et al., 2014; Hansen et al., 2016; Hiki et al., 2013; So et al., 2012; Vogt et al. 2014). Thus far, the IncA/C plasmid, a major carrier of *bla*_CMY-2_, has not yet been identified in wild animals. The absence of *bla*_CMY-2_-bearing IncA/C plasmids could simply reflect the limited studies on the characterization of *bla*_CMY-2_-carrying plasmids in wildlife, or might indicate that *bla*_CMY-2_-harbouring IncI1 plasmids are more successful among wildlife.

In addition to CMY-2, DHA-type AmpC β-lactamase genes have also been detected in *K. pneumoniae* from mouflons (*Ovis orientalis musimon*) in Austria, *E. coli* from hill mynah (*Gracula religiosa*) in Saudi Arabia, and *K. pneumoniae* ST11 isolates from Eurasian beaver (*Castor fiber*) in Switzerland (Hassan & Shobrak, 2015; Loncaric et al., 2016; Pilo et al., 2015). FOX-5 encoded by an IncA/C plasmid has been obtained from *K. pneumoniae* isolates in the US (Poirel et al., 2012). Furthermore, a novel variant of the ACT AmpC β-lactamase gene has been identified in an *Enterobacter cloacae* strain originating from glaucous gull (*Larus hyperboreus*) in Arctic Svalbard, Norway (Literak et al., 2014).

## CARBAPENEMASE-PRODUCING *ENTEROBACTERIACEAE* From WILDLIFE

Carbapenemase-producing *Enterobacteriaceae* isolates pose an urgent public health threat. New Delhi metallo-β-lactamase (NDM), as one of the most widespread carbapenemases, has been increasingly reported in human clinics, foods, domestic animals, and the environment worldwide (Abdallah et al., 2015; Chandran et al., 2014; He et al., 2017; Kumarasamy et al., 2010; Lee et al., 2016; Qin et al., 2014; Toleman et al., 2015; Yaici et al., 2016; Yong et al., 2009).

The first reported carbapenemase-producing bacteria in wild animals were isolated from black kites (*Milvus migrans*) in Germany (Fischer et al., 2013). Among 184 cefotaxime-resistant *Salmonella* spp. isolates, only one *Salmonella* Corvallis isolate belonging to ST1541 has shown reduced susceptibility to carbapenem, and carries the carbapenemase gene *bla*_NDM-1_ located on~180 kb IncA/C conjugative plasmid pRH-1738 (Fischer et al., 2013). The broad-host-range IncA/C plasmids are among the most predominant plasmids associated with *bla*_NDM-1_ in humans (Carattoli, 2013). Fischer et al. (2013) supposed that the *bla*_NDM-1_-bearing *Salmonella* Corvallis isolate might have originated from non-European countries and was transferred to Germany through the black kite migratory route, since *Salmonella* Corvallis was prevalent in South-East Asia and was emerging in North Africa and Nigeria, rather than in European countries. The complete sequence of plasmid pRH-1738 further confirms this hypothesis. Plasmid pRH-1738 exhibited high relatedness with plasmid pMR0211 obtained from human *Providencia stuartii* isolate in Afghanistan, but showed distinct differences from other sequenced NDM-1-IncA/C_2_ plasmids from Western countries (Villa et al., 2015). In addition, fosfomycin resistance gene *fosA3*, which has been rarely detected in Europe but is prevalent among CTX-M-encoding *E. coli* and *K. pneumoniae* isolates in Asia (i.e., China, Japan, and South Korea), has also been identified on NDM-1-producing plasmid pRH-1738. *bla*_NDM-1_ transferred with *fosA3* on IncA/C plasmid has only been described in clinical *E. coli* and *Citrobacter freundii* isolates in China (Qin et al., 2014). Taken together, these findings suggest that the origin of this plasmid might be in the Asiatic region.

Large-scale transmission of IMP-producing bacteria into wildlife was first reported in 2015. In total, 120 carbapenemase-producing *Enterobacteriaceae* of 10 species were obtained from silver gulls in Australia, mainly *E. coli* (*n*=85), carrying *bla*_IMP-4_, *bla*_IMP-38_, or *bla*_IMP-26_ (Dolejska et al., 2016). The *bla*_IMP-4_ gene has been found in 116 isolates, and is the most commonly detected gene among carbapenemase-producing *Enterobacteriaceae* isolates in human clinics in Australia (Bell et al., 2016; Sidjabat et al., 2015). *bla*_IMP-4_ in gulls is carried by various conjugative plasmids, mostly IncHI2-N plasmid type, followed by IncA/C plasmids, as well as IncL/M and IncI1, and is associated with a class 1 integron-containing *bla*_IMP-4_-*qacG*-*aacA4*-*catB3* array in most positive strains (Dolejska et al., 2016). The same array carried by IncA/C and IncL/M plasmids is also reportedly responsible for the dissemination of *bla*_IMP-4_ in clinical isolates in Australia (Espedido et al., 2008), and by the IncHI2 plasmid in *Salmonella* Typhimurium from a cat in Australia (Abraham et al., 2016). Furthermore, 19 different STs have been detected in IMP-4-producing *E. coli* isolates, including five prevalent lineages (ST216, ST58, ST354, ST167, and ST224), in which ST58, ST354, and ST167 are clinically relevant clone lineages (Ben Sallem et al., 2015; Fernández et al., 2014; Huang et al., 2016).

Although carbapenem resistance reported in wild animals is rare, the emergence of NDM-1 and IMP carbapenemases in wild birds is of concern.

## COLISTIN RESISTANCE GENE *mcr-1* IN *ENTEROBACTERIACEAE* FROM WILDLIFE

Colistin is widely applied in food-producing animals and is currently used as the last resort for treating infections caused by multi-resistant gram-negative bacteria (Kaye et al., 2016). Since the first identification of the plasmid-mediated colistin resistance gene *mcr-1* in China in 2015 (Liu et al., 2016b), it has been identified in *Enterobacteriaceae* isolates from food-producing animals, companion animals, food products, the environment, and humans worldwide (Anjum et al., 2016; Doumith et al., 2016; Hasman et al., 2015; McGann et al., 2016; Xavier et al., 2016; Zhang et al., 2016; Zurfluh et al., 2016).

The role of wild birds as reservoirs and vectors for the global distribution of *mcr-1* should be considered. Recently, *mcr-1* was described in an *E. coli* strain isolated from European herring gull (*Larus argentatus*) feces collected from the Kaunas (Lithuania) city dump (Ruzauskas & Vaskeviciute, 2016). However, the emergence of *mcr-1* in wildlife could be traced back to *E. coli* strains isolated in 2012 (Liakopoulos et al., 2016). Five extended-spectrum cephalosporin-resistant *E. coli* isolates obtained from kelp gulls in Ushuaia, Argentina in 2012 were found to carry *mcr-1* and *bla*_CTX-M-2_ (*n*=1) and *bla*_CTX-M-14_ (*n*=4) and exhibited elevated colistin MICs (4-8 mg/L). The *mcr-1* gene was located on a~57 kb IncI2 plasmid without *bla*_CTX-M_ in all five isolates. IncI2 plasmids, which have been detected in *E. coli* and *Salmonella* isolates from food, food-producing animals, and humans in China, Great Britain, the US, Venezuela, and Denmark, have been reported to be associated with the transmission of *mcr-1* (Anjum et al., 2016; Delgado-Blas et al., 2016; Doumith et al., 2016; Hasman et al., 2015; Meinersmann et al., 2016; Yang et al., 2016). Notably, four *mcr-1*-carrying isolates, which belong to ST744, have been previously described in Denmark and carry the *mcr-1*-bearing IncI2 plasmid (Hasman et al., 2015; Liakopoulos et al., 2016).

## CONCLUSIONS

Clinically relevant resistance, such as ESBL, AmpC cephalosporinase, carbapenemase, and colistin resistance, has been detected in wild animals, particularly wild birds, from distinct geographical areas. Thus, wild animals could serve as important reservoirs of resistant bacteria. Although the origin of bacterial resistance genes in wild animals remains unclear, as wildlife are not exposed to antibiotics directly, contact with sewage or animal manure might be one possibility (Wellington et al., 2013). Additionally, the potential of wild animals as vectors of resistant bacteria or genetic determinants should not be underestimated. Wildlife, especially migratory birds with their instinctive mobility, can carry resistant bacteria over long distances, even between continents; thus, this might be a new transmission route and partly responsible for the global dissemination of bacterial resistance. Contamination of food or water by wildlife is recognized as an important risk factor for the transmission of antimicrobial resistance or pathogens to food animals and humans (Greig et al., 2015).

Wild animals might play a vital role in the worldwide spread of clinically relevant pathogens or resistance genes. Pandemic ESBL-producing *E. coli* clones or plasmids shared by humans, domestic animals, and wildlife further strengthen this hypothesis. Thus, continued surveillance of multi-resistant bacteria in wild animals is warranted.
